# Safety and disease flare of autoimmune inflammatory rheumatic diseases: a large real-world survey on inactivated COVID-19 vaccines

**DOI:** 10.1136/annrheumdis-2021-221736

**Published:** 2021-11-25

**Authors:** Yong Fan, Yan Geng, Yu Wang, Xuerong Deng, Guangtao Li, Juan Zhao, Lanlan Ji, Xiaohui Zhang, Zhibo Song, Haoze Zhang, Xiaoying Sun, Dai Gao, Wenhui Xie, Hong Huang, YanJie Hao, Zhuoli Zhang

**Affiliations:** Department of Rheumatology and Clinical Immunology, Peking University First Hospital, Beijing, China

**Keywords:** Covid-19, vaccination, autoimmune diseases

COVID-19 vaccines are of great importance in reducing SARS-CoV-2 infection and severe cases. Patients with autoimmune inflammatory rheumatic diseases (AIIRDs) have been strongly recommended to be vaccinated according to the novel guidance because they are more vulnerable to SARS-CoV-2 infection.^1^ However, patients with AIIRDs were largely excluded from vaccine trials, leading to very limited data on the safety of COVID-19 vaccines. Notably, previous studies mainly focused on mRNA and adenovirus vector vaccines; however, little is known about inactivated COVID-19 vaccines that also have been authorised by WHO and widely used in several most populated countries, for instance, China, Brazil, Turkey and Indonesia.^2^ A large randomised clinical trial consisting of 40 382 participants has demonstrated two inactivated COVID-19 vaccines significantly reduced the risk of symptomatic COVID-19.^3^

We conducted a real-world survey to evaluate the safety profiles and disease flare in patients with AIIRDs who received any dose of inactivated COVID-19 vaccines in China. From 1 Aug 2021 to 15 Oct 2021, eligible participants completed a predefined 25-question-based questionnaire by invitation on social media or visiting the outpatient department. There was no restriction on the time interval from vaccination to completing the survey.

In total, 1507 adults patients with AIIRDs who received inactivated COVID-19 vaccine participated in this study (flow diagram in online supplemental figure 1). The median age of participants was 39 (IQR 31–51) years. There were 1166 (77.4%) female patients and 209 (13.9%) patients with self-identified allergic history. Systemic lupus erythematosus (SLE) (614, 40.7%) was the most common AIIRD among participants, followed by rheumatoid arthritis (RA) (434, 28.8%), Behcet’s disease (BD, 122, 8.1%), psoriatic arthritis/psoriasis (PsA/PsO) (76, 5.0%), primary Sjogren’s syndrome (74, 4.9%) and ankylosing spondylitis (44, 2.9%) (online supplemental figure 2).

10.1136/annrheumdis-2021-221736.supp1 Supplementary data

Among all participants, 450/1507 (29.9%) participants experienced adverse events (AEs) after vaccination (table 1). Local AEs, such as pain, redness or swelling at injection site, were reported to occur in 287 (19.0%) participants. Meanwhile, 260 (17.3%) patients reported systemic AEs after vaccination. Fatigue or sleepless (123, 8.2%) was the most reported systemic AE, followed by headache (82, 5.4%) and skin rash (55, 3.6%). The median time from vaccination shot to onset of AEs was 2 days. Most AEs were mild to moderate and self-limiting. Overall, 28 (1.9%) patients self-reported severe AEs. There were only three patients who were hospitalised due to serious diarrhoea, headache and cough. No one reported AE of interests or fatal AE, including myocarditis, idiopathic thrombocytopenic purpura, anaphylactic shock or death.

**Table 1:** Safety and flare data of AIIRDs after receiving inactivated COVID-19 vaccines

Variables	All AIIRDs	SLE	RA	BD	PsA/PsO	pSS
Participants (n)	1507	614	434	122	76	74
Female (n, %)	1166 (77.4%)	572 (93.2%)	342 (78.8%)	63 (51.6%)	34 (44.7%)	69 (93.2%)
Age (median, years)	39 (31, 51)	33 (27, 40)	50 (39, 60)	37 (30, 45)	46 (36, 58)	48 (39, 59)
Disease duration (median, years)	5 (2, 10)	5 (3, 10)	4 (2, 10)	6 (3, 10)	10 (3, 20)	3 (2, 5)
Allergic history (n, %)*	209 (13.9%)	127 (20.7%)	36 (8.3%)	21 (17.2%)	4 (5.3%)	6 (8.1%)
Complete two-dose vaccine (n, %)	1197 (79.4%)	436 (71.0%)	407 (93.8%)	87 (71.3%)	63 (82.9%)	62 (83.8%)
Inactivated vaccine band (n, %)						
Sinopharm	607 (40.3%)	272 (44.3%)	156 (35.9%)	59 (48.4%)	25 (32.9%)	26 (35.1%)
Sinovac	874 (58.0%)	340 (55.4%)	268 (61.8%)	62 (50.8%)	50 (65.8%)	47 (63.5%)
Others/uncertain band	26 (1.7%)	2 (0.3%)	10 (2.3%)	1 (0.8%)	1 (1.3%)	1 (1.4%)
AEs (n, %)	450 (29.9%)	232 (37.8%)	106 (24.4%)	34 (27.9%)	14 (18.4%)	24 (32.4%)
Local (n, %)	287 (19.0%)	160 (26.1%)	65 (15.0%)	19 (15.6%)	7 (9.2%)	13 (17,6%)
Systemic (n, %)	260 (17.3%)	120 (19.5%)	66 (15, 2%)	28 (23.0%)	8 (10.5%)	15 (20.3%)
Rash	55	28	13	9	2	3
Fever/chills	43	19	10	4	2	1
Headache	82	40	21	11	2	4
Fatigue/sleepless	123	57	31	14	2	7
Nausea/vomiting	26	15	10	3	0	1
Diarrhoea	10	7	0	1	1	1
Others	32	11	9	6	1	2
Side effects after first vaccine (n, %)	321/1507 (21.3%)	179/614 (29.2%)	69/434 (15.9%)	32/122 (26.2%)	10/76 (13.2%)	18/74 (24.3%)‡
Timing of onset, days (median)	2 (1, 3)	1 (1, 3)	2 (1, 3)	2 (1, 2)	1 (1, 2)	2 (1, 2)
Side effects after second vaccine (n, %)	140/1210 (11.8%)	68/436 (15.6%)	44/302 (14.6%)	9/87 (10.3%)	5/63 (7.9%)	4/62 (6.5%)‡
Timing of onset, days (median)	2 (1, 5)	1 (1, 7)	1 (1, 5)	2 (1, 3)	1 (1, 2)	2 (1, 7)
Self-reported severe AE (n, %)	28 (1.9%)	11 (1.8%)	4 (0.9%)	8 (6.6%)	0 (0%)	1 (1.4%)
Fatal AE of interest (n, %)†	0	0	0	0	0	0
Self-reported flare after vaccine (n, %)	158 (10.5%)	65 (10.6%)	41 (9.4%)	14 (11.5%)	3 (3.9%)	5 (6.8%)
Flare required treatment escalation (n, %)	53 (3.5%)	19 (3.1%)	11 (2.5%)	7 (5.7%)	1 (1.3%)	2 (2.7%)

*This question was described as ‘Have you ever been allergic to any food, drug or environmental substance etc before?’.

†Means anaphylactic shock, myocarditis, idiopathic thrombocytopenic purpura and death.

‡Three participants were not fully clear about that the side effects occured.after first or second vaccination.

AE, adverse event; AIIRDs, autoimmune inflammatory rheumatic diseases; BD, Behcet’s disease; PsA/PsO, psoriatic arthritis/psoriasis; pSS, primary Sjogren’s syndrome; RA, rheumatoid arthritis; SLE, systemic lupus erythematosus.

Flare of existing AIIRDs was reported by 158 (10.5%) participants, with requirement of treatment escalation in 53 (3.5%) patients. Joint pain (61/158, 38.6%) and swelling (31/158, 19.6%) were the most common manifestations of disease flare, followed by skin rash (27/158, 17.1%), morning stiffness (20/158, 12.7%) and febrile recurrence (14/158, 8.9%). Interestingly, the frequencies of AE and flare of AIIRDs were generally lower in inflammatory arthritis patients (RA or PsA/PsO) than those in patients with systemic AIIRDs (eg, SLE and BD) (online supplemental figure 3). Multivariable logistic analyses demonstrated that elderly, allergic history was the risk factor for disease flare of their underlying AIIRDs, while stable disease of AIIRDs was the negative predictor for self-reported disease flare only (online supplemental table 1).

10.1136/annrheumdis-2021-221736.supp2 Supplementary data

Our data confirmed the safety profiles, and for the first time demonstrated the disease flare after inactivated COVID-19 vaccination in patients with AIIRDs. Overall, 29.9% of participants experienced AEs after vaccination and no fatal AEs occurred, indicating the well tolerability of inactivated COVID-19 vaccines in AIIRDs population. Importantly, our results aligned with a large real-world study supported by European League against Rheumatism(EULAR) COVID-19 database (83% mRNA vaccines), whose vaccine-related AEs were observed in 31% of patients.^4^ Considering the possibility of over-activating immune system by adjuvanted vaccines, the stability of AIIRDs after vaccinations has been a principal concern. In this study, we found that although 1 in 10 reported a flare of disease after inactivated COVID-19 vaccination, fewer than 1 in 25 required treatment escalation. No episode of severe flare needing emergent hospitalisation was reported. Furthermore, we found elderly patients and those with allergic history were more likely to have disease flare after vaccinations. These call for important clinical needs for early warning of flare and close monitoring after vaccination. The incidence of AEs and AIIRD flares was generally comparable among all COVID-19 vaccines.^4–6^ These may provide evidence for rheumatologists in critical discussion on vaccine acceptance.
