# Supplementary material for: Safety and disease flare of autoimmune inflammatory rheumatic diseases: a large real-world survey on inactivated COVID-19 vaccines
Source: Ann Rheum Dis. 2021 Nov 25;81(3):443–5. doi: 10.1136/annrheumdis-2021-221736 (PMC8862022; doi:10.1136/annrheumdis-2021-221736)
Supplement: Supplementary data [file annrheumdis-2021-221736supp001.pdf]

Supplementary Figures

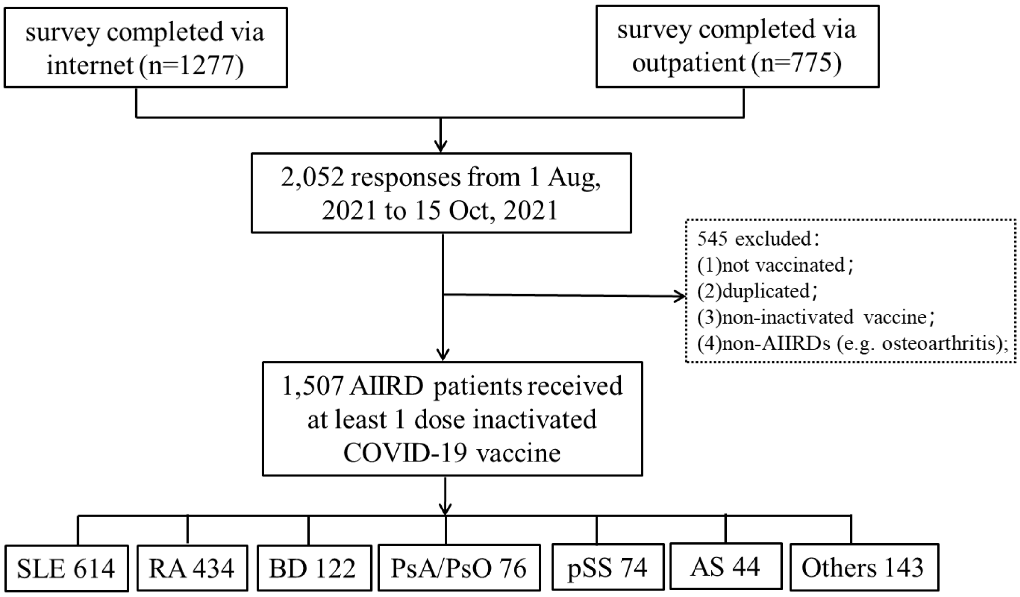

Supplementary Figure 1. Flow diagram of this study.

Abbreviations AIIRDs: autoimmune inflammatory rheumatic diseases, SLE: systemic lupus erythematosus, RA: rheumatoid arthritis, BD: Behcet's disease, PsA/PsO: psoriatic arthritis/psoriasis, pSS: primary Sjogren's syndrome, AS: ankylosing spondylitis.

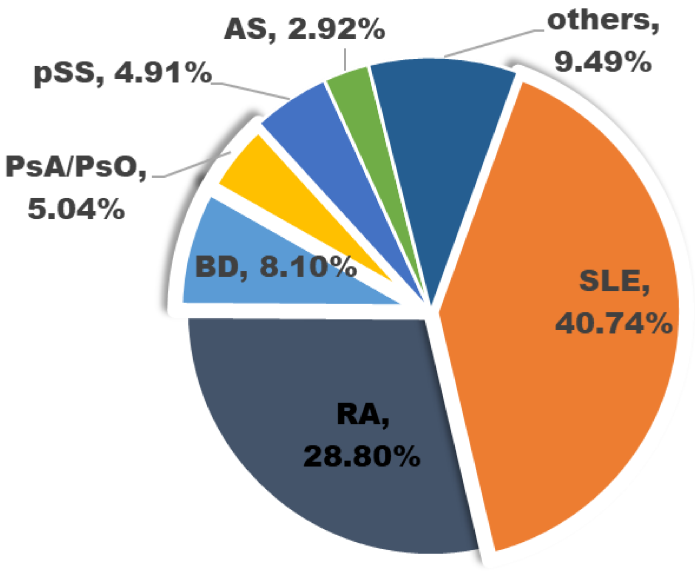

Supplementary Figure 2. Autoimmune inflammatory rheumatic diseases in 1,507 patients.

Abbreviations AIIRDs: autoimmune inflammatory rheumatic diseases, SLE: systemic lupus erythematosus, RA: rheumatoid arthritis, BD: Behcet's disease, PsA/PsO: psoriatic arthritis/psoriasis, pSS: primary Sjogren's syndrome.

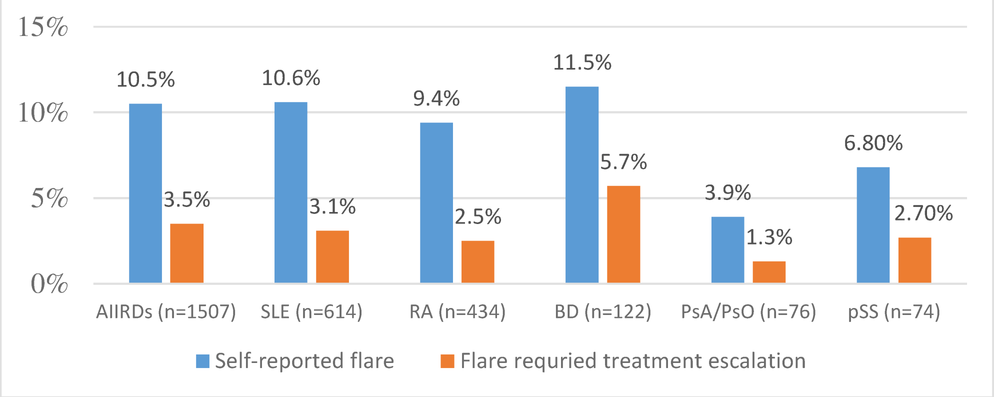

Supplementary Figure 3. Flare rates of autoimmune inflammatory rheumatic diseases (AIIRDs) after inactivated COVID-19 vaccination.

Abbreviations AIIRDs: autoimmune inflammatory rheumatic diseases, SLE: systemic lupus erythematosus, RA: rheumatoid arthritis, BD: Behcet's disease, PsA/PsO: psoriatic arthritis/psoriasis, pSS: primary Sjogren's syndrome.
