# Supplementary material for: Safety and disease flare of autoimmune inflammatory rheumatic diseases: a large real-world survey on inactivated COVID-19 vaccines
Source: Ann Rheum Dis. 2021 Nov 25;81(3):443–5. doi: 10.1136/annrheumdis-2021-221736 (PMC8862022; doi:10.1136/annrheumdis-2021-221736)
Supplement: Supplementary data [file annrheumdis-2021-221736supp002.pdf]

Supplementary Table 1. Univariable and multivariable logistic analyses for predicting disease flare of underlying AIIRDs

**Variables**	**Model 1 (self-reported flare)**				**Model 2 (treatment-escalating flare)**			
	Univariate analysis		Multivariate analysis		Univariate analysis		Multivariate analysis	
	OR (95% CI)	p value	OR (95% CI)	p value	OR (95% CI)	p value	OR (95% CI)	p value
Gender (male vs female)	0.747 (0.490-1.140)	*0.176*	/	/	0.779 (0.387-1.568)	*0.484*	/	/
Age (per 1 year)	1.001 (0.989-1.012)	*0.924*	**1.021 (1.004-1.038)**	***0.016***	1.004 (0.985-1.024)	*0.652*	**1.031 (1.006-1.058)**	***0.017***
Disease duration (per 1 year)	1.024 (0.996-1.053)	*0.098*	/	/	1.006 (0.960-1.054)	*0.81*	/	/
Allergic history	**2.420 (1.632-3.590)**	***0.001***	**2.228 (1.438-3.450)**	***0.001***	**2.750 (1.493-5.067)**	***0.001***	**2.248 (1.157-4.367)**	***0.017***
Disease category (SLE or not)	1.052 (0.753-1.469)	*0.767*	/	/	0.706 (0.399-1.251)	*0.706*	/	/
Disease category (RA or not)	0.856 (0.588-1.244)	*0.414*	/	/	0.773 (0.393-1.518)	*0.454*	/	/
Stable disease	**0.343 (0.174-0.678)**	***0.002***	**0.440 (0.215-0.902)**	***0.025***	1.079 (0.254-4.573)	*0.918*	/	/

Footnote:
1. Univariate and multivariate logistic regression analysis (stepwise backward method) was performed to investigate potential factors associated with self-reported flare and treatment-escalating flare in AIIRDs patients after inactivated COVID-19 vaccines.
2. Independent variables including gender, age, disease duration of AIIRDs, positive for allergic history, diagnosis of SLE, diagnosis of RA and self-reported stable phase of their underlying AIIRDs. Dependent variable in model 1 is self-reported flare while model 2 is treatment-escalating flare.
